# General anesthesia for repair of omphalocele in a pair of conjoined twins in Enugu, Nigeria

**DOI:** 10.4103/1658-354X.71579

**Published:** 2010

**Authors:** H. A. Ezike, V. O. Ajuzieogu, A. O. Amucheazi, S. O. Ekenze

**Affiliations:** *Department of Anesthesia, University of Nigeria Teaching Hospital, Enugu, Nigeria*; 1 *Department of Paediatric Surgery, University of Nigeria Teaching Hospital, Enugu, Nigeria*

**Keywords:** *Anesthesia*, *Nigeria*, *omphalocele*, *omphalopagus*, *surgery*

## Abstract

Conjoined twins have been viewed with fascination since antiquity. There are numerous reports in the literature documenting anesthetic management strategies for the separation of conjoined twins. There are also reports in the literature detailing anesthetic approaches for surgical procedures not involving separation. This is the first report of the anesthetic management of a set of omphalagous presenting for palliative repair of omphalocele in Nigeria.

## INTRODUCTION

Conjoined twins have been viewed with fascination since antiquity. Interest has ranged from suspicion and fear of the birth being an omen of impending disaster to exhibitionism and more recently as a subject of intense media interest.[1] The twins are monozygotic, monoamniotic, monochorionic, and are classified according to the prominent site of connection. The term “pagos” means “that which is joined or fixed.” This suffix is preceded by the anatomical site of conjunction; for example thoracopagus means joined at the thorax. World literature reflects that thoracopagus twins are the most common comprising 40–45%, omphalopagus 33%, pygopagus (sacrococcygeal junction) 19%, ischiopagus 6%, and craniopagus 2%.[2] Omphalopagus twins usually lie face to face and are joined from the xiphisternum to the umbilicus but may vary from extremely complex conjunction of a thoraco-omphalo-ischiopagus to a simple abdominal connection. An omphalocele is often present, and may rupture during delivery. There is usually a bridge of hepatic tissue as well as a peritoneal connection. The upper intestine is usually separate, but they may have a common terminal ileum and colon, with an imperforate anus. The urogenital systems show a variety of abnormalities and conjunctions.[2,3]

The exact incidence of conjoined twins is unknown, but the estimated incidence is 1 in 50 000 live births. The rate in the United States is 1 in 100 000 births. Increased incidences range from 1: 14 000 to 1: 25 000 in Asia, India, Pakistan, Thailand, and in Africa, especially East Africa, Nigeria and South Africa.[4–6] There are numerous reports in the literature documenting anesthetic management strategies for the separation of conjoined twins.[7–13] There are also reports in the literature detailing anesthetic approaches for surgical procedures not involving separation, surgical separation where one or both twins had congenital heart disease, and surgical procedures on the heart before separation.[14–17] This is the first report of the anesthetic management of a set of omphalagous presenting for palliative repair of omphalocele in Nigeria.

## CASE REPORT

**Figure 1:**
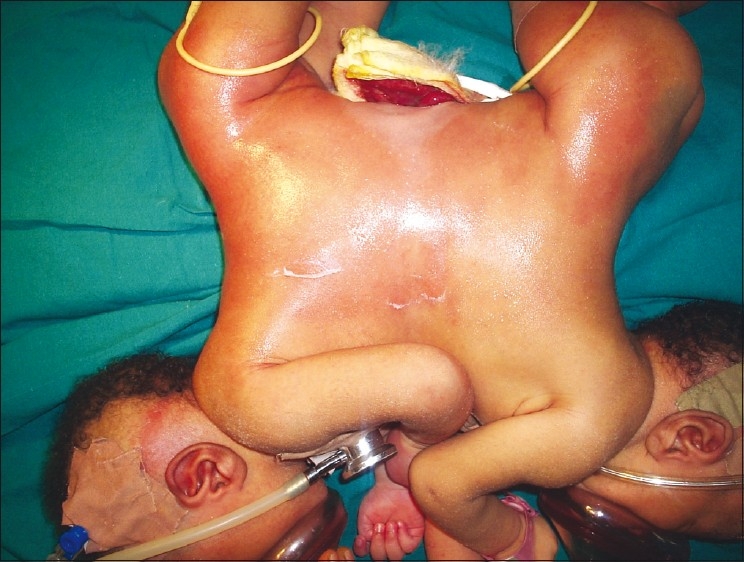
Preoperative picture showing omphalocoele

We report the case of a pair of twins joined at the upper abdomen (omphalopagus) who were delivered at a peripheral hospital by cesarean section and referred to the University of Nigeria Teaching Hospital as an emergency [Figure 1]. Together they weighed 6.0 kg. On examination, they were active, not pale and not in any obvious distress. Their chest was clinically clear and they had two separate hearts as the first and second heart sounds were heard distinctly in each twin. They were fused at the abdomen and facing each other. At the inferior aspect of the union on the abdomen was intra-abdominal content covered by peritoneum. A CT scan was requested for but could not be done due to financial constraints. An assessment of ASA IIE was made and they were scheduled for an immediate repair of the omphalocele after resuscitation. A caudal block was considered but there were technical difficulties with positioning and hence, a general anesthetic was planned.

Prior to induction of anesthesia we tested for cross circulation between twins. Glycopyrrolate was given intravenously to twin B causing an immediate increase in heart rate. However, there was no change in twin A’s heart rate for a 5 min interval following the administration of the glycopyrrolate. With no evidence of cross circulation between the twins, we felt it unlikely that twin A would become apnoeic shortly after drug administration to twin B. We evaluated our theatres to determine how best to position equipment that would be duplicated (e.g., anesthesia machine, anesthesia cart, suction, and monitors), and we color coded all the monitoring cables, breathing circuits and intravenous lines to avoid the potential for confusion about what was going to whom.

**Figure 2:**
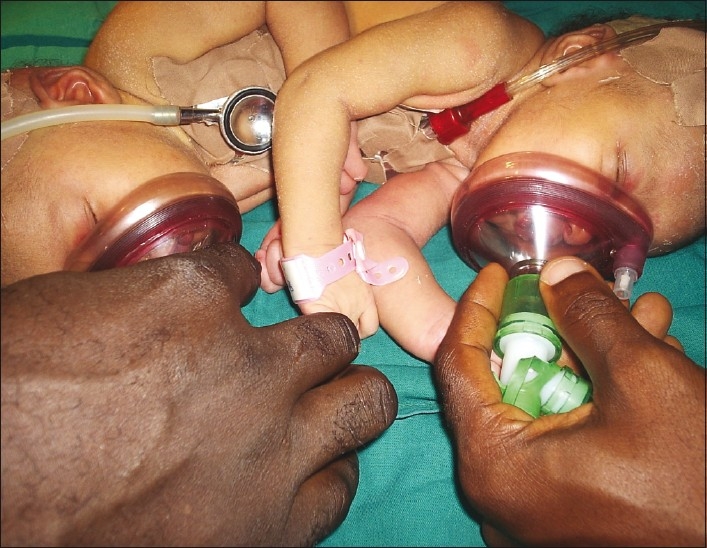
Anesthesising the twins

**Figure 3:**
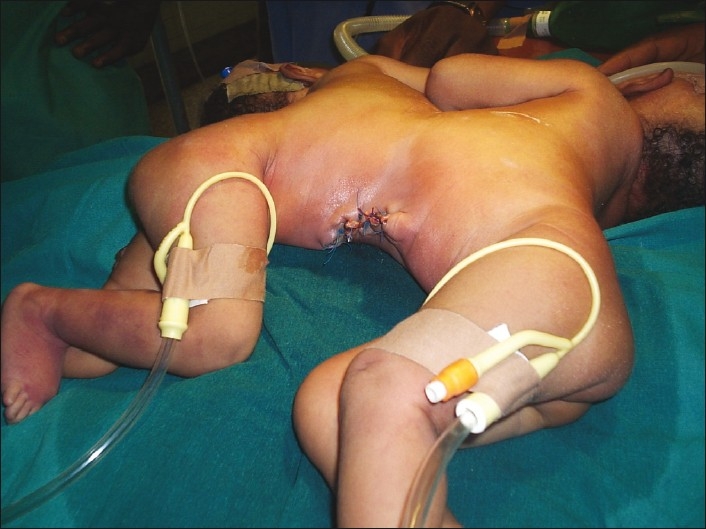
Immediate postoperative picture

Two anesthetic machines and anesthesia teams were available [Figure 2]. The theatre was kept at a thermoneutral temperature while the babies were brought in with their incubator. Standard monitors were used for both patients. Dextrose, 4.8% in saline, 0.2% was infused at maintenance rate of 4 ml/kg/h (routine pediatric maintenance); plus colloids 10–15 ml kg/h for third space losses through a peripheral IV line in each twin. The twins were positioned on their sides. Inhalation induction was performed in sequence. Each twin had to be positioned on the side breathing 50% oxygen while the other was induced. Anesthesia and analgesia was maintained with intravenous ketamine at 1.5 mg/kg. Both babies ventilated spontaneously with a guedel airway size 00 in place via a facemask. They were intubated sequentially on their side. Suxamethonium 5 mg was administered intravenously to each twin. They were intubated with a size 3 mm uncuffed endotracheal tube. The correct placement of the tube was confirmed by auscultation of both lungs for equal breath sounds and with a capnograph. Their respiration was assisted by manual ventilation via a Marpleson F circuit delivering 100% oxygen at 4 L/min. The anesthesia was uneventful with rapid recovery at the end of the surgery which lasted 45 min. Vital signs remained stable for the duration of the surgery and both twins tolerated the procedure well [Figure 3.]

## DISCUSSION

Anesthetizing conjoined twins involves three major anesthetic considerations. First, two anesthesia teams need to be available, cooperating closely in a limited workspace with clearly separate and duplicate equipment and at times interfering with each other. Second, the degree and clinical significance of crossover between the two circulations must be considered. Drugs administered to one twin may have effects on another twin. Intravenous drugs can be administered on a combined weight basis and the relative size. Testing the cross-circulation between twins can be performed by administering drugs such as glycopyrrolate to one twin and detecting the effect on the other twin.[18]

Finally, care appropriate to the surgical procedure must be provided in this unique setting. Most published reports concern anesthesia for the separation of conjoined twins, including the use of combined general with epidural anesthesia.[7] Warming the operating room to 28°C for induction ensures normothermia while the lines are being inserted. Once the drapes are in place, the temperature may be lowered. Two sets of anesthesiologists, one for each infant, are essential, as each infant has to be separately monitored throughout the procedure.[1] Essential monitoring including electrocardiogram, pulse oximetry, capnography, and urinary output is necessary. All drugs and intravenous fluids are calculated on a total weight basis with half being delivered to each twin. Because of the cross-circulation, drugs given intravenously may have an unpredictable effect. Thus, particular care is essential when administering drugs such as opioids, which should be given incrementally.

## CONCLUSION

Anesthesia for procedures on conjoined twins is a demanding, exacting, and meticulous exercise, whether prior to or during separation. Anesthesia forms part of the multidisciplinary management of the babies. Emphasis is laid on preoperative assessment, goal-directed planning of theatre and the staff involved in the surgery, duplication of all equipment necessary for anesthetizing and monitoring two infants in one operating room, and having plans in place to avoid overcrowding. Challenges encountered in anesthesia for these twins include identifying anatomical conjunctions, airway management, acquiring vascular access, the potential for enormous blood loss, and maintaining normothermia. Planning for the postsoperative period and the reconstruction and rehabilitation of the babies is essential from the time of their initial admission. Meticulous attention to detail, monitoring and vigilance are mandatory. Successful management of conjoined twins relies on close communication and cooperation of all members of the multidisciplinary team.
